# A Request

**Published:** 1853-12

**Authors:** 


A Requst.—We wish this Journal to exert a directly favorable influence upon the Medical Profession in that portion of the country which it is designed to represent, and whose wants it was originated to supply. We earnestly wish and endeavor that those who take it may come to regard it a welcome and valuable visitant, and we wish that it may bring out the talent of its patrons, and that scores of able writers may speak out the thoughts too long unuttered, and report the medical cases of interest, too long allowed to pass unrecorded. We wish our pages to be a living picture of the Medical intelligence of our time and country, and we shall gladly welcome, from our numerous subscribers, any articles calculated to throw light upon disease, to deepen or enlarge the solid principles of Medical Science.

Let private cases be reported, let diseases and epidemics of various localities be published and discussed, and let every such means of mutual self-enlightenment be faithfully employed. Let every physician thus become a center of Medical intelligence—a point of ossification, so to speak, and quackery will soon enough be at a discount.

